# Molecular and neurological features of MELAS syndrome in paediatric patients: A case series and review of the literature

**DOI:** 10.1002/mgg3.1955

**Published:** 2022-04-26

**Authors:** Lydia M. Seed, Andrew Dean, Deepa Krishnakumar, Poe Phyu, Rita Horvath, Pooja Devi Harijan

**Affiliations:** ^1^ School of Clinical Medicine University of Cambridge Cambridge UK; ^2^ Department of Clinical Neuroscience University of Cambridge Cambridge UK; ^3^ Department of Histopathology Cambridge University Hospitals NHS Foundation Trust Cambridge UK; ^4^ Department of Paediatric Neurosciences Cambridge University Hospitals NHS Foundation Trust Cambridge UK; ^5^ Department of Clinical Neuroradiology Cambridge University Hospitals NHS Foundation Trust Cambridge UK

**Keywords:** encephalopathy, genetics, lactic acidosis, m.3243A>G, MELAS syndrome, mitochondrial disease, paediatric neurology, stroke‐like episodes

## Abstract

**Background:**

Mitochondrial encephalomyopathy, lactic acidosis and stroke‐like episodes (MELAS) syndrome is one of the most well‐known mitochondrial diseases, with most cases attributed to m.3243A>G. MELAS syndrome patients typically present in the first two decades of life with a broad, multi‐systemic phenotype that predominantly features neurological manifestations––stroke‐like episodes. However, marked phenotypic variability has been observed among paediatric patients, creating a clinical challenge and delaying diagnoses.

**Methods:**

A literature review of paediatric MELAS syndrome patients and a retrospective analysis in a UK tertiary paediatric neurology centre were performed.

**Results:**

Three children were included in this case series. All patients presented with seizures and had MRI changes not confined to a single vascular territory. Blood heteroplasmy varied considerably, and one patient required a muscle biopsy. Based on a literature review of 114 patients, the mean age of presentation is 8.1 years and seizures are the most prevalent manifestation of stroke‐like episodes. Heteroplasmy is higher in a tissue other than blood in most cases.

**Conclusion:**

The threshold for investigating MELAS syndrome in children with suspicious neurological symptoms should be low. If blood m.3243A>G analysis is negative, yet clinical suspicion remains high, invasive testing or further interrogation of the mitochondrial genome should be considered.

## Graphical abstract

MELAS syndrome is one of the most well described mitochondrial diseases and typically presents during childhood. Patients display a broad, multi‐systemic phenotype with predominantly neurological manifestations, however, marked phenotypic variability has been observed, which creates a significant clinical challenge. Here we report three new clinical cases of paediatrics patients and a review of 114 paediatric cases in the literature, and discuss the clinical characteristics, histopathological features and molecular causes of MELAS syndrome in children, as well as putative molecular mechanisms of phenotypic variability observed in this mitochondrial disorder.

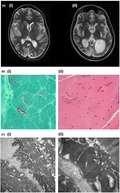

## 1 INTRODUCTION

Mitochondrial diseases are characterised by impairment of the oxidative phosphorylation (OXPHOS) pathway, which generates adenosine triphosphate (ATP) (Schon et al., 2020). Vital components of this pathway are encoded by the mitochondrial and nuclear genomes; mitochondrial diseases can arise from mutations in either mitochondrial DNA (mtDNA) or nuclear DNA (nDNA). Mitochondrial diseases can, therefore, exhibit any pattern of inheritance: maternal transmission attributable to mtDNA mutations; autosomal or X‐linked inheritance of nDNA mutations; and de novo mutations can arise in either genome (Gorman et al., 2016).

Mitochondrial diseases are largely driven by a chronic state of energy deprivation as dysfunctional mitochondria are unable to generate sufficient ATP to meet the energy demands of cells (Gorman et al., 2016). These diseases are clinically heterogeneous––due to the ubiquitous nature of mitochondria they can affect energy‐dependent processes in almost any tissue in the body (Gorman et al., 2016). Typically, tissues with a higher energy demand are most affected. Accordingly, mitochondrial diseases are among the most common causes of inherited metabolic and neurological disease (Gorman et al., 2016; McFarland et al., 2010).

The clinical phenotype of patients with mtDNA mutations is further influenced by heteroplasmy––the presence of a mixture of mutated and wild‐type mitochondrial genomes within individual cells that arises from the random distribution of mitochondria in mitotic segregation (Schon et al., 2020). The ratio of mutant to wild‐type mtDNA influences the extent to which a cell, and indeed organ, displays the disease phenotype (Schon et al., 2020). The clinical heterogeneity among mitochondrial diseases poses a significant challenge to reach a diagnosis (Lucy Raymond et al., 2018).

As next‐generation sequencing of DNA from patients' blood samples has become the first‐line diagnostic tool (Lucy Raymond et al., 2018), we must ensure that causative mtDNA variants are not missed due to low heteroplasmy in blood. Therefore, in some cases, immunohistochemical and genetic analysis of muscle biopsy may still be needed.

Certain clinical and molecular characteristics associated with mitochondrial diseases can be aggregated into discrete syndromes (Schon et al., 2020). One of the most well‐described mitochondrial diseases is mitochondrial encephalomyopathy, lactic acidosis and stroke‐like episodes (MELAS). MELAS syndrome was first described in 1984 when Pavlakis et al. reported a series of patients with this triad of clinical features (Pavlakis et al., 1984). The first molecular cause of MELAS syndrome was established in 1990––a point mutation of adenine to guanine at the highly conserved position m.3243 on the mitochondrial genome, in the *MT‐TL1* gene (OMIM *590050) (Goto et al., 1990; Kobayashi et al., 1990; McKusick‐Nathans Institute of Genetic Medicine, 2022). This mutation is responsible for ~80% of MELAS syndrome cases (Goto et al., 1990), and most remaining causative mutations occur at other loci in the mitochondrial genome.

More than 16 in 100,000 individuals carry the m.3243A>G mutation (Majamaa et al., 1998), yet MELAS syndrome has a prevalence of only 0.18 per 100,000 (Yatsuga et al., 2012). Carriers of m.3243A>G are clinically heterogeneous. Their phenotypes constitute a broad spectrum ranging from asymptomatic to being affected by one of the several diseases, such as maternally inherited diabetes and deafness (MIDD) (van den Ouweland et al., 1992), chronic progressive external ophthalmoplegia (CPEO) (Sotiriou et al., 2009) and MELAS syndrome.

Clinical diagnostic criteria for MELAS syndrome were first published in 1992 (Hirano et al., 1992). Key features included: a stroke‐like episode before 40 years‐of‐age; encephalopathy characterised by seizures or dementia; and lactic acidosis or ragged‐red fibres (RRFs) on muscle biopsy. More recently published diagnostic criteria stratifies patients depending on the presence of clinical features of stroke‐like episodes and evidence of mitochondrial dysfunction (Yatsuga et al., 2012).

## 2 METHODS

### 2.1 Ethical compliance

Patients and their family members have given consent to the study ‘Genotype and phenotype in inherited neurological disease’ (REC number 13/YH/0310).

### 2.2 Case series

**FIGURE 1:**
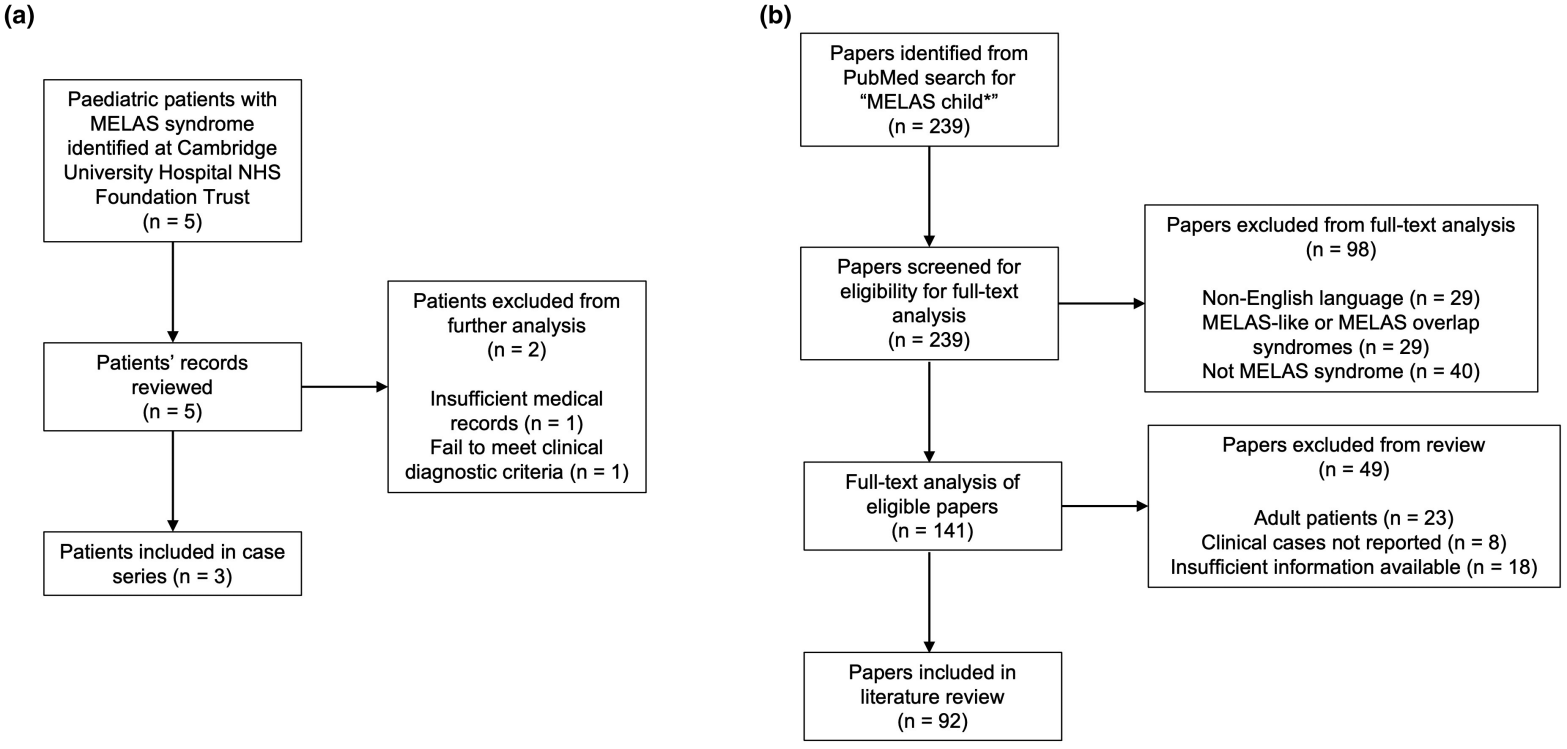
PRISMA flow diagrams showing the selection of paediatric MELAS syndrome cases from (a) Cambridge University Hospital NHS Foundation Trust included in the case series, and (b) the PubMed search included in the literature review

A retrospective analysis of paediatric patients with MELAS syndrome registered under Cambridge University Hospitals NHS Foundation Trust was performed. Patients were identified by emailing all paediatric neurology and clinical genetics consultants. A total of five patients were initially identified (Figure 1a). Two patients were excluded from the case series: one patient had been identified as an m.3243A>G carrier upon cascade screening following a diagnosis of MIDD in their maternal grandmother and was asymptomatic; insufficient medical records were available for the other patient (Figure 1a). Medical letters, inpatient notes, genetics sequencing reports, brain MRIs, and muscle biopsy histology slides were reviewed for the three patients.

### 2.3 Literature review

A comprehensive literature review of case reports indexed in PubMed of paediatric patients with MELAS syndrome was performed. The term “MELAS child*” was searched and the article type filter set to case reports only. The search yielded 239 results (Figure 1b). PubMed entries that did not report clinical cases, provided insufficient information, were not written in the English language, reported adult patients, or reported patients with MELAS‐like syndromes, MELAS overlap syndromes or a disease that was not MELAS syndrome were excluded from further analyses (Figure 1b). The molecular and neurological features of 114 paediatric patients with MELAS syndrome were reported from a full‐text analysis of 92 papers.

## 3 RESULTS

### 3.1 Case series

Here, we present a series of three paediatric patients with MELAS syndrome (Table 1).

**TABLE 1:** Summary of the clinical features of MELAS syndrome patients in the case series upon initial presentation

Case	Mutation	Heteroplasmy		Age (years)	Seizures	Myopathy	Lactate		Headache	Visual disturbance	Gastrointestinal complications
		Blood	Muscle				Plasma (0.6–2.5)	CSF (1.1–2.2)			
1	m.3243A>G	58%	NM	15	+	−	2.7	4.5	+	+	−
2	m.3955G>C	13%	82%	8	+	−	2.9	NM	+	−	+
3	m.3243A>G	NM	‘High levels’	6	+	+	Raised	NM	−	+	+

Abbreviation: NM, not measured.

#### 3.1.1 Case 1

**FIGURE 2:**
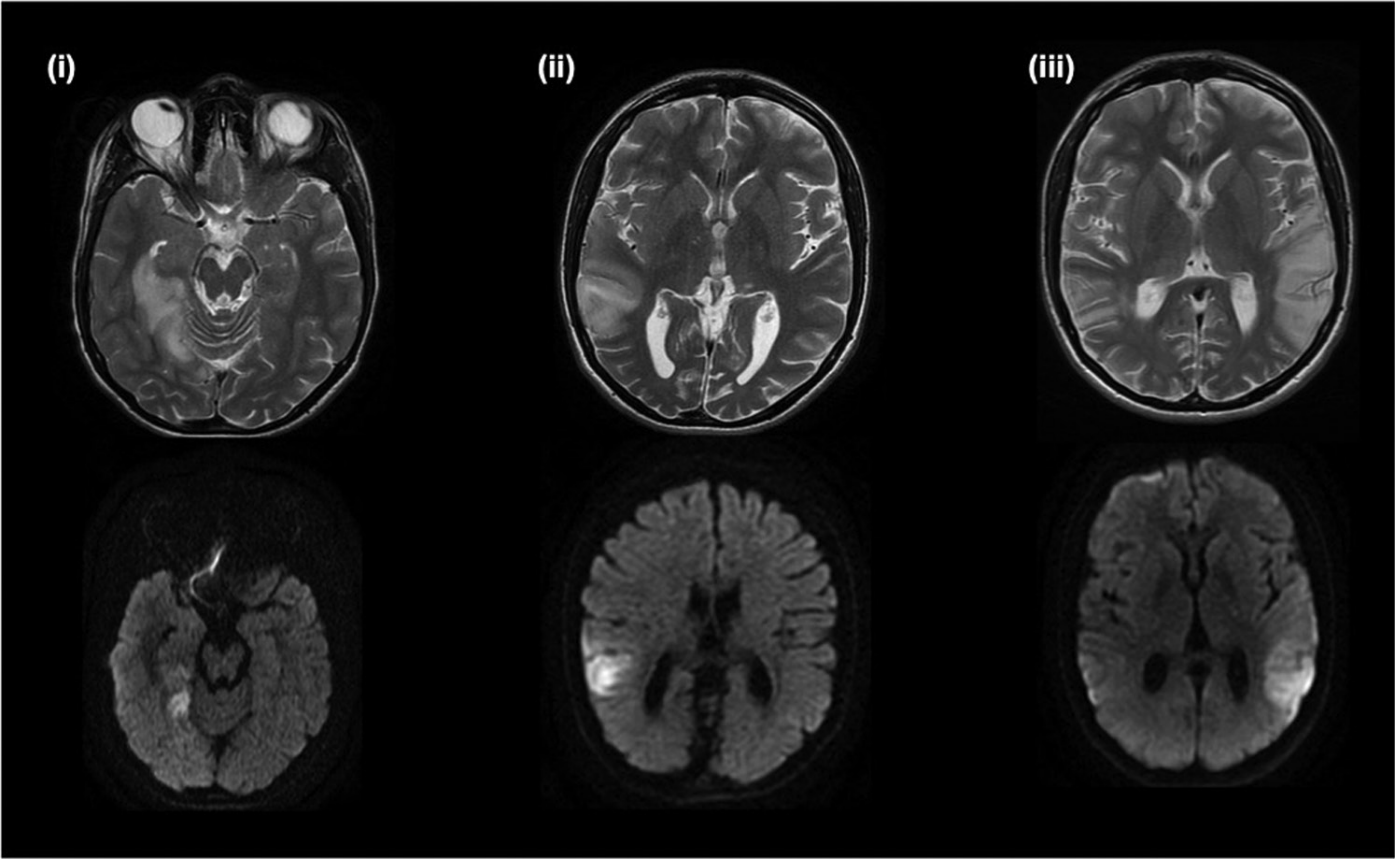
Brain MRIs, axial view T2‐weighted (first row) and DWI (second row), showing development and resolution of signal abnormalities within the (i) right medial temporal lobe upon initial presentation, (ii) right lateral temporal lobe 4 months after initial presentation, and (iii) left lateral temporal lobe 5 months after initial presentation

A 15‐year‐old male presented with a 5‐day history of headache and 1‐day history of vomiting, on a background of coordination difficulties, short stature, sensorineural hearing loss and borderline hypertension. Early development was normal. During admission his headaches worsened, and he had episodes of unilateral vision loss and eye deviation with head tilt. Electroencephalogram showed subclinical focal seizures and serial brain MRIs showed development and resolution of signal abnormalities within the right medial, right lateral temporal and left lateral temporal lobes over 6 months (Figure 2). Inpatient targeted genetic testing for m.3243A>G and common pathogenic *POLG* mutations (OMIM *174763) (McKusick‐Nathans Institute of Genetic Medicine, 2022) revealed m.3243A>G was present at 58% heteroplasmy. Cascade screening in the patient's mother was performed, but the mutation was not detected in maternal blood or skeletal muscle samples.

#### 3.1.2 Case 2

**FIGURE 3:**
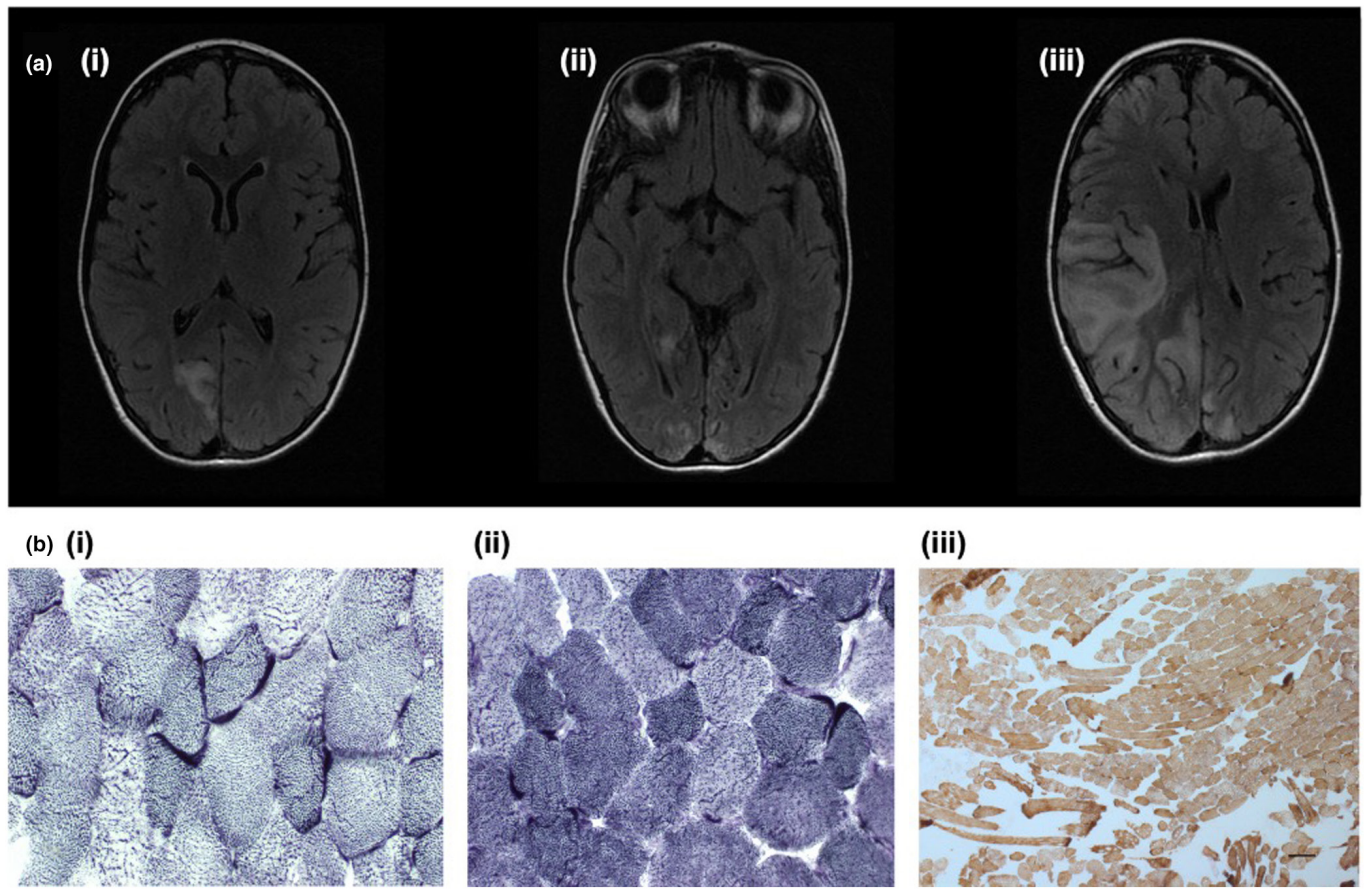
(a) Brain MRI axial FLAIR sequences showing development signal abnormalities within (i) right occipital lobe on admission, (ii) that progressed to the left occipital lobe 5 months later, and (iii) further progressed to the right temporal, insula and frontal lobes a further 6 months later. (b) Histology slides of biopsy from left quadriceps demonstrating subsarcolemmal accentuation upon (i) SDH and (ii) NADH staining (×40 magnification), and (iii) no obvious COX‐negative‐SDH‐positive muscle fibres upon sequential COX‐SDH staining

An 8‐year‐old female with normal early development presented with headache, vomiting and focal seizures that responded to intravenous (IV) lorazepam. Initial brain MRI revealed signal abnormalities in the right occipital lobe (Figure 3ai). Whole genome sequencing (WGS) (reporting threshold 15%), which included the mitochondrial genome, returned no abnormalities and there was no family history of MELAS syndrome. Mitochondrial DNA sequencing from blood, urine and skeletal muscle samples detected a novel *MT‐ND1* variant (m.3955G>C) at 13% heteroplasmy in the blood, 46% in urine and 82% in muscle (OMIM *516000) (McKusick‐Nathans Institute of Genetic Medicine, 2022). Muscle fibre biopsy displayed mitochondrial proliferation––a compensatory mechanism in mitochondrial cytopathies (Phadke, 2017)––demonstrated by subsarcolemmal accentuation upon SDH and NADH‐TR staining (Figure 3bi, Figure 3bii). Upon COX‐SDH staining, there was an absence of COX‐negative SDH‐positive fibres (Figure 3biii).

#### 3.1.3 Case 3

**FIGURE 4:**
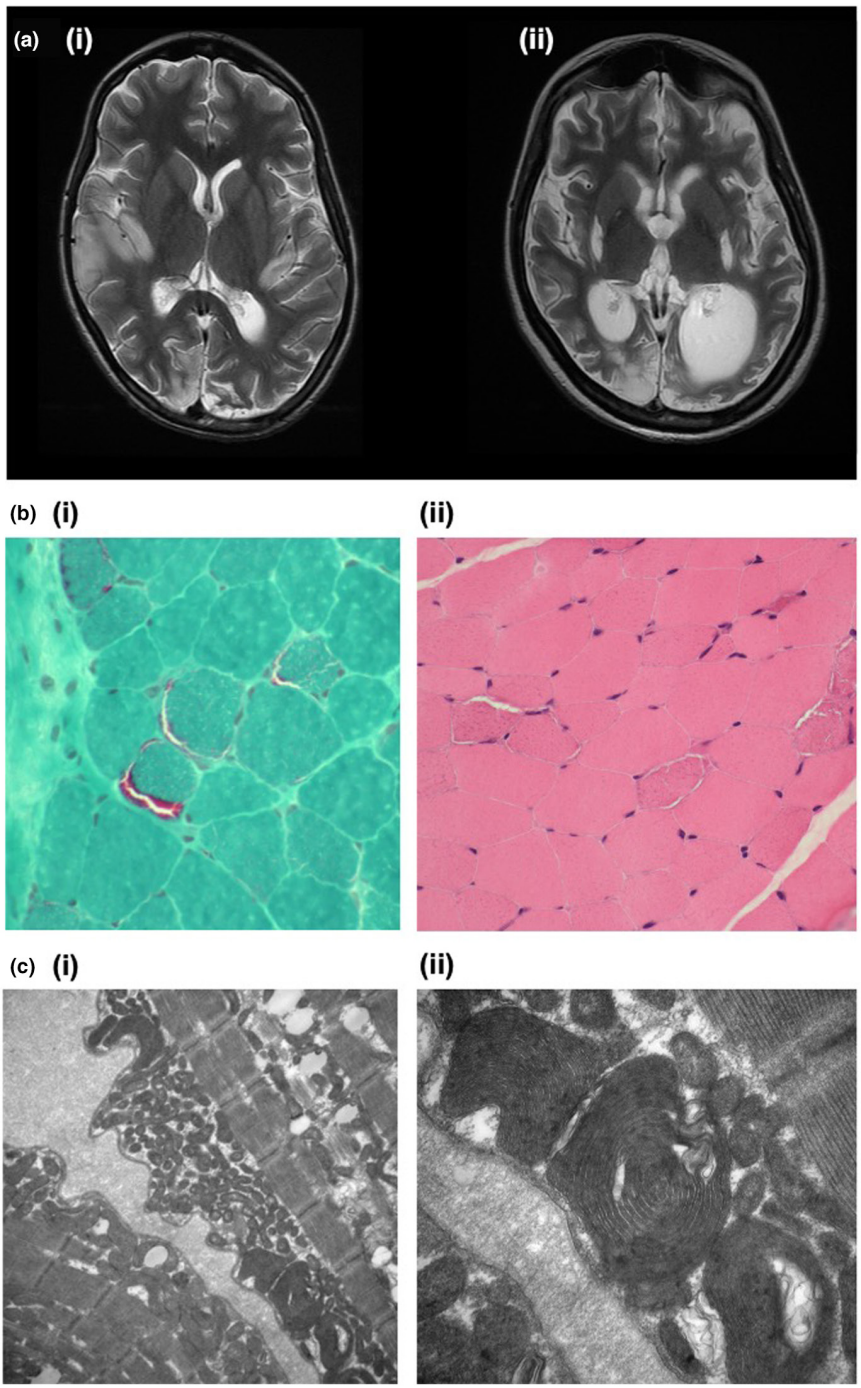
(a) Brain MRI T2‐weighted sequences showing progressive generalised atrophy and reciprocal dilatation of the ventricular system over 5 years: (i) 4 years after initial presentation; (ii) 9 years after initial presentation, following second emergency surgery. Multiple old signal abnormalities are present in bilateral basal ganglia, bilateral occipital lobes and right insula. (b) Histology slides of a muscle biopsy: (i) subsarcolemmal Gomori positivity forming the RRFs characteristic of mitochondrial cytopathy (×60 magnification); (ii) H&E staining showing muscle fibres, with a clear surrounding rim, that vary in size, as well as basophilic stippling (×40 magnification). (c) Electron microscopy at (i) ×15,000 magnification and (ii) ×40,000 magnification, both showing subsarcolemmal mitochondria with abnormal forms

A 6‐year‐old female presented with proximal myopathy, lactic acidosis, neurodevelopmental delay, epilepsy and vision loss. The classical RRFs were visualised on muscle biopsy (Figure 4bi) and the H&E‐stained section showed a few basophilically stippled fibres (Figure 4bii), indicative of pathological RRFs (Niu et al., 2017). A few fibres stained very weakly for cytochrome oxidase (data not shown). Abnormal mitochondria was visualised on electron microscopy (Figure 4c). After no common pathogenic *POLG* mutations were detected, sequencing of the entire mitochondrial genome was performed. This revealed m.3243A>G at ‘high levels’ in the muscle, although there was no family history of conditions associated with this mutation. Aged 15 years, she developed recurrent intestinal pseudo‐obstruction and underwent emergency jejunostomy refashioning but deteriorated post‐operatively with suspected bowel perforation and intra‐abdominal sepsis that required a terminal loop ileostomy. Post‐operatively, brain MRI showed severe generalised cerebral volume loss, secondary to encephalomalacia (Figure 4aii). She developed multi‐organ failure, cardiovascular shock and severe lactic acidosis, and died.

### 3.2 Literature review

A total of 114 paediatric patients with MELAS syndrome were identified in the literature. The mean age of presentation was 8.1 years (SD = 4.1), ranging from 4 months to 17 years. All cases in the literature described clinical features of stroke‐like episodes. However, in 11% of cases, transient deficits in neurological function were simply described rather than explicitly labelled and reported as a ‘stroke‐like episode’.

The most prevalent neurological manifestation of stroke‐like episodes was epilepsy or seizures, which were present in 84% of cases. Headaches, weakness, and visual disturbances were each reported in 62% of cases. Half of the cases in the literature displayed developmental delay (50%), and just over one‐quarter reported hearing deficits (26%). All seven neurological features were present in only six cases.

The m.3243A>G mutation was reported in 54% of cases, and mutations within *MT‐TL1* accounted for over two‐thirds (67%) of cases altogether. Heteroplasmy levels were reported in 42% of cases: blood heteroplasmy in 38 cases and muscle heteroplasmy in 30. Heteroplasmy was reported in a range of other tissues, namely fibroblasts (*n* = 16) and urine (*n* = 11) as well as hair, buccal mucosa and nails, among others, in a minority of cases. In one post‐mortem report, heteroplasmy was measured in 10 different organs (Stenqvist et al., 2005).

The highest heteroplasmy reported in blood was of the m.3271T>C mutation at a level of 84.3% (Brisca et al., 2015). Three studies reported homoplasmic or near‐homoplasmic levels of m.12146A>G (Calvaruso et al., 2011), m.5541C>T (Hatakeyama et al., 2015), and m.3243A>T (Longo et al., 2008) in muscle. Homoplasmy of m.12146A>G was also reported in fibroblasts (Calvaruso et al., 2011). Heteroplasmy levels were reported in both blood and muscle or other tissues for 30 cases. A higher heteroplasmy in a tissue other than blood was detected in 87% of cases. Full details of the molecular and neurological features of paediatric patients with MELAS syndrome reported in the literature are available in Supplementary File S1.

## 4 DISCUSSION

MELAS syndrome typically presents in childhood, with the majority of patients presenting in the first two decades of life (Hirano & Pavlakis, 1994; Pavlakis et al., 1984), at a mean age of 8.1 years (SD = 4.1), and a second peak after age 40 years (El‐Hattab et al., 2015).

The defining clinical features of MELAS syndrome are neurological. Stroke‐like episodes typically manifest with seizures (84%), visual disturbances (62%), motor weakness (62%), and are often accompanied by a migrainous headache (62%), often with vomiting (El‐Hattab et al., 2015; Ng et al., 2019; Ohno et al., 1997). One‐fifth of paediatric patients displayed all four of these neurological features. Typically, there is a short‐term recovery following a stroke‐like episode, but neurological deficits accumulate over time and progress to dementia. Additionally, 26% of cases in the literature had sensorineural hearing loss. In contrast in adults this has been reported in up to 75% of patients (Majamaa et al., 1998), beginning mildly but progressing insidiously (Sproule & Kaufmann, 2008).

Normal early development was noted to be a ‘cardinal manifestation’ of MELAS syndrome in the original diagnostic criteria (Hirano et al., 1992). Hirano et al.’s analysis of 69 adult and paediatric cases found that 91% had normal early development. However, among the paediatric patients reviewed in the literature, prior to presenting with stroke‐like episodes, only 50% of patients had reached appropriate developmental milestones.

Cortical involvement has often been described as asymmetric, although a recent retrospective study observed symmetric lesions more frequently, in up to one third of patients (Bhatia et al., 2020). Despite the apparent sparing of the frontal lobe, executive function deficits have been observed, suggesting that an additional diffuse neurodegenerative or metabolic process is contributing to this cognitive impairment (Kraya et al., 2019; Sproule & Kaufmann, 2008). While lactic acid accumulation (Iizuka et al., 2002), microvascular pressure (Faraci & Heistad, 1990) and ion concentration (Muñoz et al., 2015) are believed to be involved, the underlying mechanism of the stroke‐like episodes has not yet been established. On neuroimaging, the cortical and subcortical signal abnormalities do not correspond to the classical vascular territories, hence the term ‘stroke‐like’. They predominantly involve the temporal, parietal and occipital cortices (Figures 2 and 3a), most commonly affecting the primary visual cortex, middle‐third of the primary somatosensory cortex, and the primary auditory cortex––regions of high neuronal density and metabolic demand (Bhatia et al., 2020). In the acute setting, it can be challenging to differentiate MELAS syndrome from an acute ischaemic stroke. This distinction is vital to inform therapeutic decisions––the management of acute ischaemic stroke involves thrombolysis which would be inappropriate in MELAS syndrome. A scoring criterion based on vessel signs from fluid attenuated inversion recovery (FLAIR) images has recently been developed to clinically differentiate between these two similar clinical presentations (Chong et al., 2021).

Peripheral sensorimotor neuropathy is a common, yet often subclinical, manifestation of MELAS syndrome (Kaufmann et al., 2006). It typically affects the lower limbs distally and is progressive. Nerve conduction studies have found mixed axonal loss and demyelinating sensorimotor neuropathy to be the most common polyneuropathy affecting patients with m.3243A>G (Kärppä et al., 2003). Older male patients are more likely to develop this.

Myopathy, although non‐specific, is another one of the hallmark characteristics of MELAS syndrome. Exercise intolerance and muscle weakness are also often observed, as well as motor developmental delay in a small proportion of patients (Sproule & Kaufmann, 2008). In a cohort of m.3243A>G mutation carriers––who either had MELAS syndrome or were oligosymptomatic––histochemical testing and exercise physiology data revealed an inverse correlation between muscle mutational load and maximal oxygen uptake and workload (Jeppesen et al., 2006).

Cardiomyopathy is observed in a large proportion of MELAS syndrome patients. Nonobstructive, concentric hypertrophy is most typically reported, although dilated and hypertrophic cardiomyopathy are also often found (Sproule & Kaufmann, 2008). Left ventricular hypertrophy has been reported in ~40% of MELAS syndrome patients (Anan et al., 1995) and a positive linear correlation between the level of m.3243A>G heteroplasmy and left ventricular mass index has also been identified (Majamaa‐Voltti et al., 2002). Additionally, cardiac conduction abnormalities, namely Wolff–Parkinson–White syndrome, appear to have a prevalence up to four times higher among patients with MELAS syndrome and m.3243A>G compared to the normal population, with some reports of these symptoms even manifesting earlier than the neurological phenotype (Sproule et al., 2007).

Serum plasma lactate was raised in all patients in the case series, and CSF lactate was very high in the one patient in whom it was measured (Table 1). As a consequence of the inability of dysfunctional mitochondria to generate sufficient ATP via the OXPHOS pathway, there is a shunting of pyruvate to lactate which manifests systemically as lactic acidosis (Sproule & Kaufmann, 2008). Thus, lactic acidaemia is a clinical manifestation of mitochondrial cytopathy and is not specific for MELAS syndrome.

The mutation m.3243A>G has long been associated with diabetes mellitus––it causes MIDD (van den Ouweland et al., 1992), and diabetes mellitus was included in the initial description of MELAS syndrome (Pavlakis et al., 1984). Diabetes mellitus is a common systemic feature of MELAS syndrome in children, and is present in up to one third of all patients (Sproule & Kaufmann, 2008; Yatsuga et al., 2012). It can be type 1 or type 2 in nature and manifests at a mean age of 38 years (El‐Hattab et al., 2015).

Another systemic manifestation of MELAS syndrome is growth failure––short stature has been reported in between 33% and 82% of individuals. This may be explained by an observed association between the m.3243A>G mutation and growth hormone deficiency (Yorifuji et al., 1996) or may reflect the chronic state of systemic energy‐deprivation (Sproule & Kaufmann, 2008). Other reports of endocrine dysfunction have occasionally been reported in MELAS syndrome patients, namely hypothyroidism, hypoparathyroidism and hypogonadotropic hypogonadism (El‐Hattab et al., 2015).

The m.3243A>G mutation is associated with features of gastrointestinal smooth muscle dysfunction such as constipation, diarrhoea, gastric dysmotility, intestinal dysfunction and intestinal pseudo‐obstruction (Fujii et al., 2004). Moreover, likely as a consequence of the gastrointestinal smooth muscle dysfunction, failure to thrive has been reported in over a quarter of children with MELAS syndrome (Yatsuga et al., 2012).

Rarer manifestations of MELAS syndrome affecting other organ systems have been described. Reported renal manifestations include focal segmental glomerulosclerosis, nephrotic proteinuria and renal insufficiency (Alcubilla‐Prats et al., 2017; Mochizuiki et al., 1996). Rarely, dermatological manifestations have been observed; vitiligo was found in 11% of patients (Karvonen et al., 1999). One case report describes pulmonary artery hypertension in a patient with MELAS syndrome, although it is unclear whether this is a true association (Sproule et al., 2008).

The majority of MELAS syndrome cases are caused by m.3243A>G within the *MT‐TL1* gene, which encodes tRNA^Leu(UUR)^. This mutation was found to be responsible for 54% of paediatric cases in the literature, which is considerably lower than the previously reported rate of ‘more than 80%’ of all cases of MELAS syndrome (Sproule & Kaufmann, 2008). This may be a reflection of a higher level of genetic heterogeneity among paediatric MELAS syndrome patients but is more likely due to publication bias––rarer causes of MELAS syndrome are more noteworthy and therefore more likely to be published in the literature.

Over two‐thirds of paediatrics cases were attributed to a mutation in *MT‐TL1*. Most remaining causes of MELAS syndrome are due to other mtDNA point mutations that occur in genes encoding either other tRNAs or respiratory chain complex subunits (Table 2). In addition to mutations in the mitochondrial genome, variants in nuclear genes that encode mitochondrial enzymes have also been reported to cause MELAS syndrome; notably, variants in the mtDNA polymerase gamma gene, *POLG1*, are the commonest nuclear cause (Deschauer et al., 2007; Hikmat et al., 2017).

**TABLE 2:** A comprehensive list of the variants in the mitochondrial genome reported in the literature and online databases as pathogenic for MELAS syndrome

Gene	Locus type	Variants associated with MELAS syndrome	References
*MT‐TF*	tRNA Phe	G583A, G586A, T616C	Mitomap, Landrum et al. (2008)
*MT‐TV*	tRNA Val	A1630G, G1606A, G1642A, G1644A	Mitomap (2008)
*MT‐RNR2*	16S rRNA	C3093G	Sproule and Kaufmann (2008)
*MT‐TL1*	tRNA Leu (UUR)	A3243G, A3243T, G3244A, A3251G, A3253G, G3255A, C3256T, T3258C, A3260G, 3271delT, T3271C, A3274G, T3291C, A3302G, C3303T	Mitomap (2008)
*MT‐ND1*	NADH dehydrogenase subunit 1 (complex I)	T3308C, G3376A, G3380A, G3481A, G3697A, G3946A, T3949C	Mitomap (2008)
*MT‐TI*	tRNA Ile	G4298A	Landrum et al. (2008)
*MT‐TQ*	tRNA Gln	G4332A	Mitomap
*MT‐TM*	tRNA Met	G4450A	Mitomap
*MT‐TW*	tRNA Trp	G5521A, 5536insT, G5538A, G5540A, C5541T, T5543C	Landrum et al. (2008), Mitomap
*MT‐TA*	tRNA Ala	G5591A	Landrum et al. (2008)
*MT‐TN*	tRNA Asn	T5728C	Landrum et al. (2008)
*MT‐CO1*	Cytochrome c oxidase subunit 1, (complex IV)	A7445G	Landrum et al. (2008)
*MT‐TS1*	tRNA Ser (UCN)	7471insC, G7497A, T7511C, T7512C	Landrum et al. (2008)
*MT‐TK*	tRNA Lys	T8316C, A8344G, T8356C, T8362G, G8363A	Landrum et al. (2008)
*MT‐ATP6*	ATP synthase 6	G8969A	Landrum et al. (2008)
*MT‐CO3*	Cytochrome c oxidase subunit 3 (complex IV)	T9957C	Sproule and Kaufmann (2008)
*MT‐ND3*	NADH dehydrogenase, subunit 3 (complex I)	T10158C	Mitomap
*MT‐ND4*	NADH dehydrogenase, subunit 4L (complex I)	A11084G	Sproule and Kaufmann (2008)
*MT‐TH*	tRNA His	A12146G, G12147A	Mitomap
*MT‐TL2*	tRNA Leu (CUN)	G12276A, A12299C, G12315A	Landrum et al. (2008)
*MT‐ND5*	NADH dehydrogenase, subunit 5 (complex I)	A12770G, G13042A, A13045C, A13084T, T13094C, G13513A, A13514G	Mitomap (2008)
*MT‐ND6*	NADH dehydrogenase, subunit 6 (complex I)	G14453A	Sproule and Kaufmann (2008)
*MT‐TE*	tRNA Glu	T14674C, T14709C, G14710A, G14739A	Landrum et al. (2008)
*MT‐CYB*	Cytochrome b (complex III)	14787del4, T14864C	Sproule and Kaufmann (2008), Emmanuele et al. (2013)
*MT‐TT*	tRNA Thr	G15915A	Landrum et al. (2008)
*MT‐TP*	tRNA Pro	G15967A	Landrum et al. (2008)

Detection of a mutation associated with MELAS syndrome not only accelerates the speed of diagnosis, but also eliminates the need for further invasive, and expensive investigations. For example, in Case 1, following the detection of m.3243A>G at 58% heteroplasmy in the blood by targeted sequencing, a genetic cause of MELAS syndrome was established and no further invasive investigations were required. Targeted testing for this mutation is a high‐yield first‐line investigation, given that it is responsible for most cases of MELAS syndrome. If negative, the mitochondrial genome and *POLG* should be interrogated for pathogenic mutations by applying a large next‐generation panel assay to exome sequencing or WGS data (Lucy Raymond et al., 2018; Schon et al., 2020).

However, if the detection threshold of the assay is higher than the potential level of heteroplasmy in the blood, further testing is required to reach a diagnosis, as demonstrated in Case 2. A novel mutation in *MT‐ND1* was initially undetected by WGS because the reporting threshold for calling variants was lower than the level of heteroplasmy in blood. Only upon targeted sequencing of mtDNA from the blood sample, that employed more sensitive sequencing techniques, was this novel mutation detected and a diagnosis reached. Retrospective analysis of WGS data confirmed the variant. This highlights the importance of pursuing a molecular diagnosis by further investigating mtDNA variants with low blood heteroplasmy rates, and possibly with a muscle biopsy, if a patient displays a phenotype suggestive of MELAS syndrome despite having a negative trio WGS. Moreover, a family history of MELAS syndrome is only present in around one‐quarter of patients (Hirano et al., 1992) and it was not present in any patients in the case series herein, further emphasising the clinical value of performing thorough genomic investigations.

Patients with MELAS syndrome can display markedly different clinical features with variable disease progression (Table 1). Mutations at different loci in the mitochondrial, and even nuclear, genomes can cause MELAS syndrome; locus heterogeneity may account for some of the clinical heterogeneity observed. For example, m.3243A>G is the causative mutation in Cases 1 and 3, whereas Case 2 has a novel pathogenic variant in a different mtDNA gene. A notable difference in clinical features is the presence of visual disturbances in Cases 1 and 3, but not in Case 2 (Table 1). This could be attributed to the different molecular aetiologies.

Furthermore, striking phenotypic heterogeneity is observed among patients with the same pathogenic mutation. Case 3 presented at 6 years of age with proximal myopathy, epilepsy, vision loss and neurodevelopmental delay, whereas Case 1 presented at 15 years of age with headaches and vomiting, on a background of coordination difficulties, hearing impairment and renal disease. At 15 years, Case 3 developed gastrointestinal complications which progressed to multi‐organ failure, cardiovascular shock and severe lactic acidosis, and died, whereas Case 1 has displayed no gastrointestinal abnormalities. There are several molecular mechanisms that may contribute to phenotypic variability.

The heteroplasmy level of m.3243A>G has been shown to correlate with disease burden (Grady et al., 2018). The different heteroplasmy levels of mitochondria with the pathogenic mutation in affected organs likely contribute to phenotypic variation––a higher burden of mutant mitochondria confers a more severe phenotype in that tissue. However, as demonstrated by Case 2 and the literature review, blood heteroplasmy does not always reflect heteroplasmy in affected organs, such as skeletal muscle. Heteroplasmy levels were higher in tissues other than blood in 87% of paediatric cases in the literature; heteroplasmy in affected organs cannot be reliably predicted from blood heteroplasmy. Although, due to the constraints of sampling affected organs, it is challenging to fully appreciate and ascertain the role of heteroplasmy levels in contributing to the variable phenotypic expression among patients with MELAS syndrome.

Recent research has highlighted the potential role of nuclear genomic variants in modulating the phenotypes observed in patients with the same pathogenic m.3243A>G mutation (Pickett et al., 2018). Whole exome sequencing (WES) performed on a series of MELAS syndrome patients with the classical m.3243A>G mutation revealed pathogenic mutations in several nuclear genes associated with aspects of the MELAS syndrome phenotype (Chakrabarty et al., 2021), such as sensorineural hearing loss, diabetes and cardiomyopathy, suggesting that nuclear genetic background may modify the MELAS syndrome phenotype observed. The additive effect of nuclear polymorphisms on the MELAS syndrome phenotype remains to be fully explored.

Mutations in the nuclear genome may also influence the penetrance mtDNA mutations, contributing to the phenotypic variation among MELAS syndrome patients. *Uittenbogaard* et al. reported an individual with MELAS syndrome and their asymptomatic mother to both carry the m.1630A>G mutation––which occurs in the *MT‐TV* gene that encodes tRNA^val^––at near‐homoplasmic levels (Uittenbogaard et al., 2019) (OMIM *590105) (McKusick‐Nathans Institute of Genetic Medicine, 2022). Fibroblasts from the proband displayed severely impaired OXPHOS metabolism, however, OXPHOS metabolism was normal in the mother's fibroblasts. WES revealed the proband to be heterozygous for a nonsense mutation in the nuclear gene, *VARS2*, which interacts with tRNA^val^. This variant was not detected in the mother, and therefore is likely responsible for modulating the penetrance of m.1630A>G and hence the contrasting phenotypes.

In Uittenbogaard et al.’s study, transmission electron microscopy was performed which identified ultrastructural changes in chromatin between the proband and her mother, suggestive of differential epigenomic regulation that may explain the observed difference in OXPHOS metabolism (Uittenbogaard et al., 2019). Further work is needed to elucidate the role of the epigenome in contributing to MELAS syndrome, and indeed other mitochondrial diseases.

## 5 CONCLUSION

The clinical phenotype of MELAS syndrome varies greatly, as demonstrated by the case series presented herein, and there should be a low threshold for investigating this disease in children and young people presenting with a range of neurological symptoms. Robust assessment of clinical phenotype is of paramount importance in MELAS syndrome, allowing for earlier intervention and appropriate management of seizures and stroke‐like episodes.

The literature review of paediatric cases revealed the clinical presentation of MELAS syndrome to be grossly similar to that in adults, with stroke‐like episodes and seizures present in the vast majority of patients, often accompanied by headaches, visual disturbances and weakness. Notably, we found developmental delay to be more prevalent here than in the previous reports (Hirano et al., 1992). Further, hearing deficits were markedly less common in children compared to adults (Majamaa et al., 1998).

A MELAS syndrome biomarker in blood may soon be clinically validated to enable classification and monitoring of the disease––a recent study on a deeply phenotyped MELAS syndrome cohort revealed several mitochondrial biomarkers that correlate strongly with severity (Sharma et al., 2021). A growing number of mutations in the mitochondrial and nuclear genomes have been identified as pathogenic for MELAS syndrome. If clinical suspicion for MELAS syndrome remains high despite inconclusive results from WGS of blood tissue, the mitochondrial genome should be interrogated further, with a lower threshold and high sensitivity assay, and invasive testing of affected tissues, such as muscle biopsy, should also be considered.

## CONFLICT OF INTEREST

The authors declare no potential conflict of interest.

## AUTHOR CONTRIBUTIONS

Lydia M. Seed reviewed medical notes, carried out the literature review and drafted the manuscript. Andrew Dean interpreted and selected appropriate histology slides. Poe Phyu interpreted and selected appropriate brain MRIs. Deepa Krishnakumar and Pooja Devi Harijan recommended appropriate patients for the case series. Andrew Dean, Deepa Krishnakumar and Poe Phyu contributed edits to the manuscript. Rita Horvath and Pooja Devi Harijan contributed to manuscript writing.

## ETHICAL APPROVAL

Informed consent for diagnostic and research‐based studies was obtained for all subjects in accordance with the Declaration of Helsinki protocols and approved by a local institutional review board (Yorkshire & The Humber—Leeds Bradford Research Ethics Committee 13/YH/0310).

## A PATIENT CONSENT STATEMENT

The parents of all patients gave consent for publication.

## Supporting information


**Supplementary File 1** XXXXX

## Data Availability

Further data can be made available upon reasonable request to the first author.
